# Biological Aging in People Living with HIV on Successful Antiretroviral Therapy: Do They Age Faster?

**DOI:** 10.1007/s11904-023-00646-0

**Published:** 2023-01-25

**Authors:** Sara Svensson Akusjärvi, Ujjwal Neogi

**Affiliations:** The Systems Virology Lab, Division of Clinical Microbiology, Department of Laboratory Medicine, Karolinska Institutet, Stockholm, Sweden

**Keywords:** People living with HIV, Antiretroviral therapy, Biological aging, Immunometabolism

## Abstract

**Purpose of Review:**

In the absence of a prophylactic/therapeutic vaccine or cure, the most amazing achievement in the battle against HIV was the discovery of effective, well-tolerated combination antiretroviral therapy (cART). The primary research question remains whether PLWH on prolonged successful therapy has accelerated, premature, or accentuated biological aging. In this review, we discuss the current understanding of the immunometabolic profile in PLWH, potentially associated with biological aging, and a better understanding of the mechanisms and temporal dynamics of biological aging in PLWH.

**Recent Findings:**

Biological aging, defined by the epigenetic alterations analyzed by the DNA methylation pattern, has been reported in PLWH with cART that points towards epigenetic age acceleration.

**Summary:**

The hastened development of specific clinical geriatric syndromes like cardiovascular diseases, metabolic syndrome, cancers, liver diseases, neurocognitive diseases, persistent low-grade inflammation, and a shift toward glutamate metabolism in PLWH may potentiate a metabolic profile at-risk for accelerated aging.

## Introduction

Without a prophylactic or therapeutic vaccine and cure, the most amazing achievement in the battle against HIV was the discovery of effective, well-tolerated combination antiretroviral therapy (cART). The successful administration and adherence of cART have transformed this deadly virus infection into a manageable, lifelong, chronic disease. Even so, successful long-term treatment of HIV is associated with an increased and accentuated development of non-AIDS-related pathologies [1]. People living with HIV (PLWH) on successful treatment do not only die at an earlier age compared to uninfected individuals, but they also show signs of a higher incidence of age-related disorders due to the chronic inflammatory environment and immune cell activation [2]. The exact reason for this is still not known. Due to the globally increased life span of PLWH, predictive statistical models have shown that the number of PLWH over 50 is rising. In the Dutch ATHENA cohort, the proportion of PLWH aged 50 years or older was predicted to increase from 28% in 2010 to 73% in 2030 [3]. When discussing age, the most common measurement used is an individual's chronological age, meaning the time passed from birth to a given date. However, biological aging is more important when discussing what healthy aging means for PLWH. Biological aging is defined as the function and activity of the body's innate responses to external factors like an infection. With increasing age, the performance of the immune response decreases with a decline in cell division and the development of age-associated disorders such as dementia, frailty, and cardiovascular disease. The hallmark of biological aging and the molecular biomarker of cellular aging are multifaceted. It includes telomere shortening, DNA damage-mediated genomic instability, epigenetic alterations, cellular senescence, and replication arrest. Apart from those, the accumulation of reactive oxygen species (ROS), mitochondrial dysfunctions resulting in lower energy availability, and deregulated homeostasis of proteins (proteostasis) have also been proposed [4, 5••]. Furthermore, compromised autophagy, cellular senescence due to the proinflammatory-associated secretory phenotype (SASP), altered microbiome compositions, and dysregulation of RNA processing have recently been added to the old hallmark of aging [6••]. The new comprehensive hallmarks of aging are highly relevant for PLWH can be attributed to the characteristics of low-grade chronic inflammation and immune activation despite having successful therapy. The present review aims to discuss the current understanding of the immunometabolic profile in PLWH, potentially associated with biological aging, to provide a better understanding of the mechanisms and temporal dynamics of biological aging in PLWH.

## Biological Aging with HIV

PLWH endure various age-related comorbidities compared to the general population. This has led to the hypothesis that PLWH may present higher biological age than their HIV-negative counterparts with similar chronological ages. Studies have pointed toward increased frailty [7, 8], higher incidence of cardiovascular disease [9], and malignancies [10, 11], in PLWH, compared to HIV-negative individuals of similar age. These observations led researchers to hypothesize that PLWH presumably ages faster and might need medical attention at an earlier age. However, the use of chronological age to compare aging may be highly confounded by the genetic background of the individuals, lifestyle, diet, and environmental exposures. With improved technologies, more advanced molecular data, and machine learning methods, "aging clocks" have been developed to predict the biological age more reliably. Biological aging, defined by the epigenetic alterations analyzed by the DNA methylation pattern, has been reported in PLWH, with cART pointing towards increased epigenetic aging markers in both the brain and blood [12, 13]. Moreover, a more recent study in PLWH from the NEAT001/ANRS143 cohort reported higher epigenetic age in PLWH using four different epigenetic clocks during treatment naïve conditions. The epigenetic age can be reduced by initiating antiretroviral therapy, but it remains higher in PLWH after two years of therapy compared to the HIV-negative counterparts [14••]. However, several critical factors can be attributed, including the severity of the initial immune dysfunction, exposure to the antiretrovirals, lifestyle, diet, gut microbiota dysbiosis, and persistence of low-grade inflammation. Biomarkers for transcriptomic aging can also measure biological age. Initial transcriptomic clocks of aging developed based on the RNA expression signature for age classification showed a low accuracy [15]. However, improvements are in progress [16]. Recent advancements in metabolomics data acquisition and reproduction, a metabolic signature of the aging process, and a metabolomics-based age predictor (metaboAge) have been proposed [17, 18]. The advantage of the metabolic signature of aging over the other molecular clocks is that it can guide lifestyle changes and potential interventions to improve metabolic health and immune function, leading to healthy aging and improving lifespan.

## Immunometabolic reprogramming in PLWH

Immunometabolism is a concept used to describe the interplay between metabolic processes and immune cell functions. Immunometabolism is controlled mainly by the alteration of interconnected metabolic pathways, e.g., glycolysis, the pentose phosphate pathway (PPP), the tricarboxylic acid cycle (TCA cycle), fatty acid oxidation (FAO), and fatty acid synthesis (FAS) that are regulated by amino acid metabolism. A recent review summarized the links between HIV-1 infection and immunometabolic pathways [19••]. The metabolism of cells is a determinant factor that can regulate how the stimulation affects both the phenotype and function of cells. An increased biomolecule and energy production can alter metabolic pathways, consequently modulating the overall status of the immune cell, e.g., activation state and effector functions. Cell metabolic processes are regulated by external stimuli such as T-cell receptor (TCR) stimulation, glucose and glutamate availability, growth factors, cytokines, or oxygen tension. These signals can sustain the survival and effector functions of cells by inducing catabolic (generation of ATP for energy and amino acid production) or anabolic (biomolecule production for the synthesis of nucleic acids, lipids, and proteins) processes [20]. With such a large variety of inducers comes a plethora of internal signaling pathways that mediate the downstream adaptation, including protein kinase B (AKT), phosphoinositide 3-kinase (PI3K), mammalian target of rapamycin (mTOR), hypoxia-inducible factor (HIF), cellular myelocytomatosis oncogene (c-Myc), and AMP-activated protein kinase (AMPK) signaling [21]. Additionally, as the oxygen tension ranges between 1 and 12% from body compartments to arterial blood, cellular metabolism can be aerobic or anaerobic [22, 23]. Aerobic metabolism relies on the three pillars of central carbon metabolism (CMM): glycolysis, the TCA cycle, and the PPP. Additionally, oxidative phosphorylation (OXPHOS) in the mitochondria is coupled to the TCA cycle, which results in a slow metabolic process generating high amounts of ATP. Anaerobic metabolism consists of glucose conversion into lactate, resulting in rapid ATP production but less energy-efficient [21]. The mTOR signaling pathway consists of two serine/threonine protein kinases complexes, mTORC1 and mTORC2. Of these, mTORC1 regulates lipid metabolism, mitochondrial biosynthesis, and induction of HIF signaling, while mTORC2 is believed to mainly regulate glucose uptake, glycolysis, gluconeogenesis, and OXPHOS [24, 25]. Downstream of mTOR is HIF signaling, a major adaptor to cellular stress, regulating the transcriptional activity of more than 100 downstream proteins. Activation results in the dimerization of the two isoforms of HIF, namely, HIF-1ɑ and HIF-1β. Upon dimerization, these proteins are translocated into the nuclei, where they bind the hypoxia response element (HRE), inducing transcriptional activation of target genes for cell survival, proliferation, differentiation, angiogenesis, and apoptosis [26]. Therefore, HIF is a signaling pathway mainly activated during low oxygen availability. As such, HIF signaling is the major mediator of glycolysis while decreasing the oxidation of ɑKG to succinate, leading to decreased TCA metabolites and increased citrate production that can be used for fatty acid metabolism [27]. On the other hand, during high oxygen availability, alternative pathways are induced to regulate metabolic reprogramming based on external stimuli and demand. Upregulation of the PI3K/Akt/mTOR/HIF signaling promotes anabolic metabolism, while catabolic processes are induced upon low ATP availability by inducing AMPK signaling [28]. Additionally, during activation-induced reprogramming of T cells, c-Myc has been described as essential [29]. Collectively, all these pathways create a complex network for metabolic adaptations to ensure appropriate cellular functions during homeostasis and disease.

Emerging evidence suggests that immunometabolism is crucial in HIV pathogenesis [19••]. In PLWH, the link between the processes mentioned above and pathways is not well understood. Several factors can regulate the susceptibility to HIV during infection, such as the cell’s metabolic activity, activation stage, and glycolytic enzymes, where elevated glycolysis and OXPHOS favor infection in CD4^+^ T cells [30, 31]. In a recent study from our group, we detected reduced expression of the glucose transporter (Glut1) on T lymphocytes in HIV-positive elite controllers (EC) who naturally control viral replication compared to uninfected controls [32]. The uptake of glucose through Glut1 is vital for virus production [33]. Therefore, this could be a potential mechanism restricting virus propagation in EC. When looking at latent HIV, data from a recent study suggested that the HIV reservoirs could use glutaminolysis as an alternative pathway to generate energy [34]. Glutaminolysis (where glutamine is lysed to glutamate and other TCA cycle product) is the main pathway that fuels the TCA cycle and OXPHOS in some T-cell subsets, such as naïve and memory T-cells. Another recent study reported that HIV infection is higher in T cells selected for OXPHOS activity [31]. Additionally, it has been shown that compromised metabolic steps preceding OXPHOS can accumulate lipids [34]. Further, our in vitro study observed a significant effect on latent virus reactivation when modulating glutaminolysis, indicating a substantial role of glutaminolysis in regulating latent HIV [35••]. Moreover, a recent seminal study showed how glutaminolysis is essential in senescent cells and proposed that inhibiting glutaminolysis in the aging body may prevent age-associated disorders and even extend the life span [36]. This data showed a small fraction of the complex interplay between metabolism, immune cell functions and HIV. Our recent studies indicated that immune-metabolic reprogramming is typical in long-term treated individuals in several cohorts. The metabolic rewiring during prolonged therapy is associated with the viral reservoir and linked to the dysregulation of glutamate metabolism. High extracellular glutamate levels can be involved in developing neurological disorders in PLWH [37••]. However, neurotoxicity mediated by elevated glutamate levels can be rescued by both lactate and pyruvate scavengers [38, 39]. Conclusively, immune cell metabolism is regulated by a complex network that can determine the activation and function of cells and environmental adaptations. Additionally, in the context of HIV, oxidative stress and metabolic alterations can contribute to a chronic inflammatory environment in PLWH.

Specifically in HIV infection, studies have shown how PLWH on ART both have increased surface expression of Glut1 and increased markers of aerobic glycolysis in both CD4^+^ and CD8^+^ T cells [31, 40, 41]. Furthermore, in central and naïve CD4^+^ T cells, Glut1 expression has been correlated to the immune activation marker HLA-DR, while metabolism relying on glycolysis correlates to the mitochondrial density of cells [40, 41]. This data indicates that these cells might have a higher mitochondrial capacity making them more suited to respond to infections rapidly. However, some studies have shown how Glut1 is a marker of T-cell activation. In contrast, others have shown how Glut1 expression on CD8^+^ T cells is not associated with other activation markers [40]. Similarly to T cells, monocytes in PLWH on ART also have an elevated expression of Glut1 [42]. Therefore, if Glut1 should be viewed as a marker of the activation of cells remains unknown.

Furthermore, in PLWH, HIV infection has been associated with mitochondrial dysfunction from plasma metabolomics data. Specifically, this study showed how PLWH had a decrease in mitochondrial fatty acid oxidation (β-oxidation) and enrichment of oxidation in the smooth endoplasmic reticulum (ER) (Ω-oxidation) [43]. These augmented mitochondrial functions can contribute to aberrant immune cell functions in PLWH. Additionally, the viral protein Vpr can contribute to a dysregulation of glutamate metabolism in infected macrophages [44]. After preferential glutaminolysis, the mTORC1 signaling pathway promotes cell growth while inhibiting autophagy [45]. In monocytes, studies have shown how the HIV reservoir is sustained by the enrichment of pyruvate metabolism and glycolytic pathways for PPP if key mitochondrial enzymes are downregulated [46]. Moreover, there seems to be a shift in infected macrophages from OXHPOS upon quiescence, as they mainly rely on glutaminolysis and glucose and fatty acid metabolism [34]. Additionally, the memory capacity of monocytes mainly relies on glycolysis mediated through Akt/mTOR/HIF signaling [47, 48]. Collectively, these studies have contributed to our understanding of how metabolism shapes the activity and function of cells during HIV infection. However, further studies must elucidate how these factors relate to chronic inflammation and earlier aging in PLWH.

Appropriate cellular adaptation of immune cells to their environment partially depends on metabolic reprogramming. Simultaneously, viral evolution has developed strategies allowing the exploitation of the host cellular metabolic machinery to promote virus replication and spread in the body [49]. Studies on HIV infection have shown how the metabolic activity of a cell plays an essential role in virus susceptibility, where CD4^+^ T cells with high glycolysis and OXPHOS have a higher permissiveness to infection [30, 31, 50]. This metabolic profile is similar to activated T cells, while both naïve and resting T cells are less susceptible to infection [51, 52]. Another factor that increases both the susceptibility and transcription of HIV is high glucose availability, mediated by a ROS-induced uptake of glucose through Glut1 and upregulation of HIF-1ɑ and CXCR4 [33, 53]. Just as important as the metabolic environment is for HIV susceptibility, it also shows after the virus has infected a cell. Viral replication is a highly exhausting process where the virus needs to take over both the cellular biosynthesis machinery and processes for the biosynthesis of macromolecules [54]. During HIV infection, these energy requirements are met by increasing glycolysis through the upregulation of glucose receptors in both monocytes and lymphocytes [40, 42, 55–57]. The virus can also regulate glycolytic enzyme activity to promote replication [58]. However, studies have also shown that these glycolytic enzymes can have antiviral functions, showing a dual activity during HIV infection [59, 60]. The dependency of glycolysis in virus-infected cells also leaves options for intervention as infected cells’ viability is more sensitive towards glycolytic inhibition than uninfected cells [30]. Therefore, even as the virus increases the metabolic rate for its propagation and spread, it can open for metabolic targeting of pathways essential for virus propagation.

Besides being an essential pathway for metabolic reprogramming, studies have shown how the mTOR pathway regulates both HIV latency and memory T cell function [61–63]. In PLWH on ART, T cells rely to a great extent on glucose metabolism regulated by mTORC1. In contrast, the capacity of CD8^+^ T cells in PLWH with natural control of infection (EC) to suppress infection is regulated by mTORC2-controlled glucose metabolism and OXPHOS [64]. The enrichment of mTORC2 in EC indicates its relevance in HIV persistence [65]. Moreover, studies have shown how before the loss of natural control of infection, the metabolism of EC exhibit a shift to aerobic glycolysis, deregulated mitochondrial activity together with immune activation, and elevated oxidative stress [66]. Even as this data shows how metabolic adaptations might be involved in control/loss of natural control of infection, whether the virus’ production induces the shift in metabolism or the other way around remains a mystery.

**Fig. 1:**
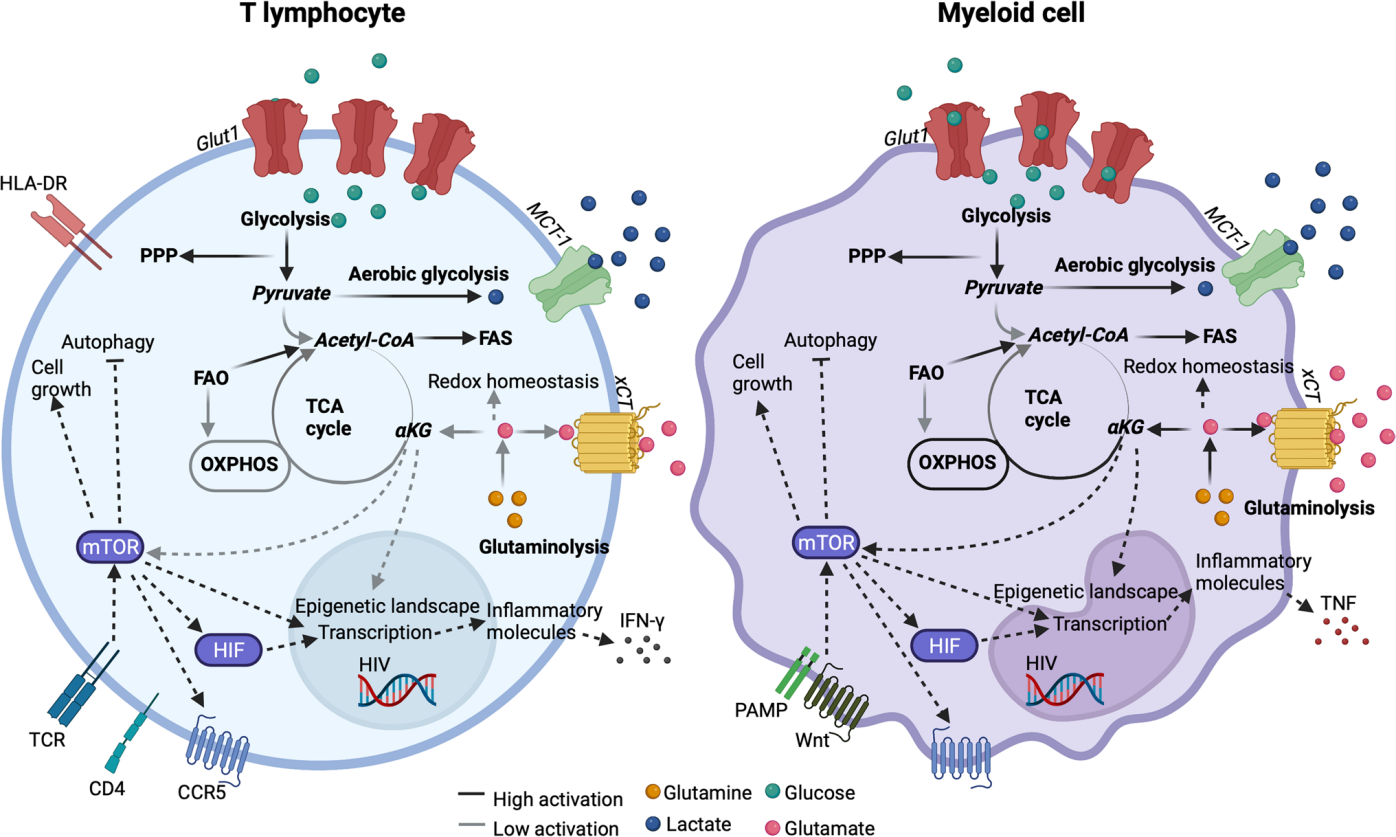
Proposed metabolic reprogramming of T cells and myeloid cells in PLWH on cART based on current literature. Black lines describe pathways with high activation, while grey lines describe pathways with low activation in the cells. This collectively gives an overview of the metabolic pathways on which the different cell types are less dependent. The metabolites glutamine, lactate, glucose, and glutamate are marked in orange, blue, green, and pink, respectively. Their relative intracellular/extracellular abundance is represented based on the localization of the metabolite in the figure. The figure was created using Biorender.com and adapted from Svensson Akusjärvi S: Immunometabolic reprogramming during suppressive HIV-1 infection. Doctoral Thesis, Karolinska Institute 2022 [86]

Glycolysis is crucial for the activation of cells. In monocytes, it is needed for TNF secretion, while it is also essential for T cells to exert effector functions, such as INF-γ production [67, 68]. The proinflammatory profile of monocytes, measured by TNF secretion, can be suppressed by lactate [67]. Another study also showed how lactate could suppress the HIV-specific functions of CD8^+^ T cells, emphasizing how single metabolites can alter immune cell functions [69]. Additionally, the epigenetic landscape of cells can be modulated by the downstream effects of αKG [70]. Even as this data shows how individual metabolites can play essential roles in the modulation of different immunometabolic processes, these are just initial studies starting to elucidate the functions of individual metabolites or signaling pathways. The dynamics of how these systems or structures act in favor or against the functions of immune cells in PLWH as a consequence of infection or alternative comorbidities require further understanding. Additionally, even as many studies have tried to elucidate what role immunometabolism in different immune cells (e.g., lymphocytes, myeloid lineage cells) plays in PLWH (Fig. 1), exactly which metabolic changes occur, and their impact on immune cell functions, the inflammatory environment, and aging remains unknown.

## System Biological Studies Defining the Immune Aging in PLWH

**Fig. 2:**
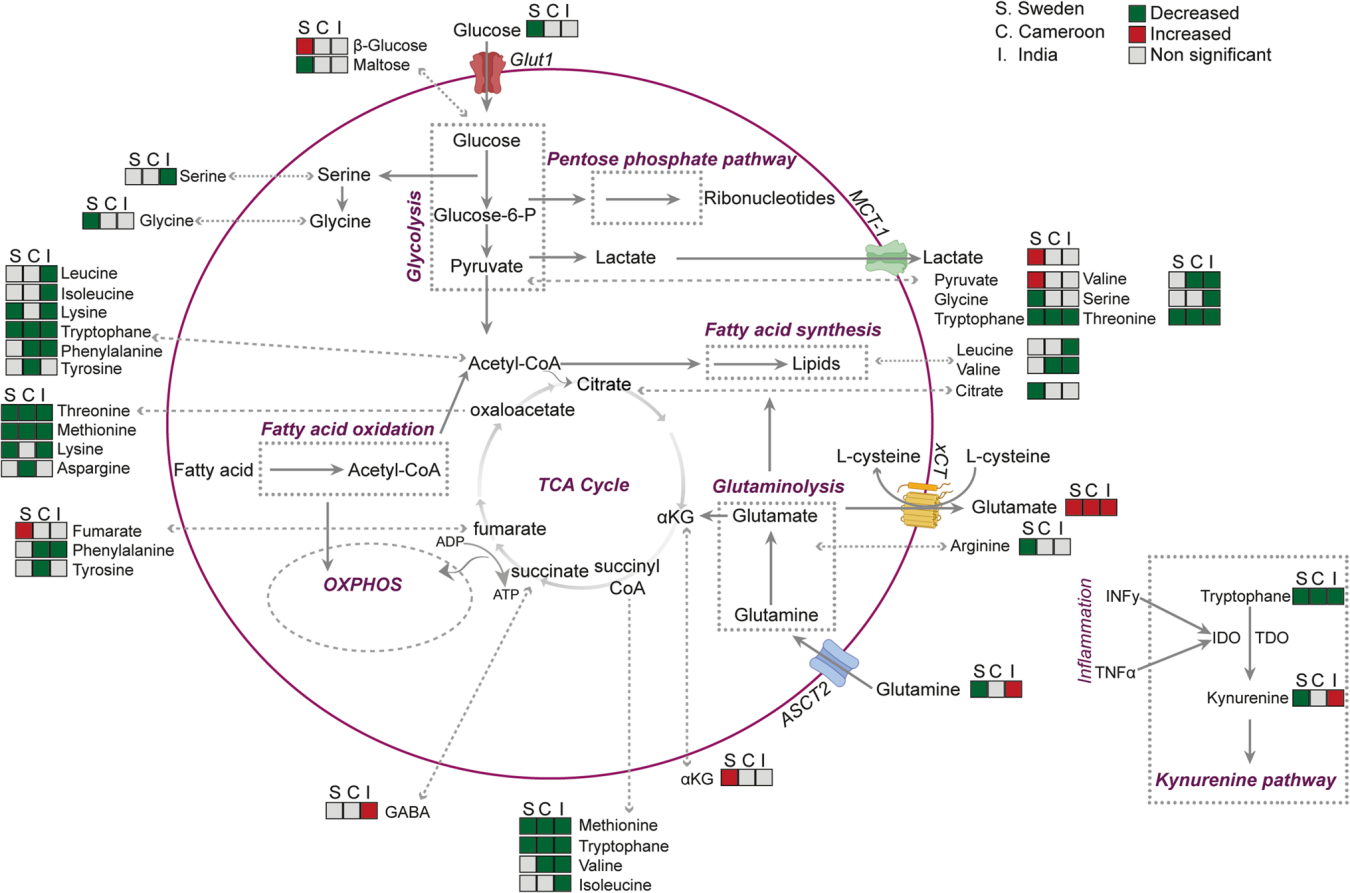
Alterations in metabolite levels in PLWH on cART compared to HC in cohorts from Cameroon (C), India (I), and Sweden (S) from targeted plasma metabolomics. Green boxes represent decreased metabolites in the PLWH on the cART group, while red boxes represent metabolites enriched at *p* < 0.05. Grey boxes represent metabolites that were not significantly different between the groups. The figure was adapted from Svensson Akusjärvi S: Immunometabolic reprogramming during suppressive HIV-1 infection. Doctoral Thesis, Karolinska Institute 2022 [86]

Technological advancements in the big data field have rapidly developed, allowing a comprehensive snapshot of different levels of biological processes, from the genome to the metabolome. Data-driven, unbiased scientific analyses have been able to aid in our understanding of physiological aberrations during different disease conditions [71]. Multi-omics system biology analysis combining single omics-level data, such as genomics, transcriptomics, epigenome, proteomics, and metabolomics, can provide helpful information about biological processes and functions [72]. The end product, metabolites, will be a response in the form of a phenotype. Alternatively, as each step in this line will result in decreased information, a bottom-up approach can be used. However, in a bottom-up approach, a significant fraction of information will be lost along the way. Even so, this phenotype-first-driven approach can be advantageous by targeting the analysis from a higher level to find in-depth differences. Integrating data from several omics layers is a method in the system biology approach [73]. Albeit less applied in HIV infection, both single-level and integrative-omics analyses have contributed significantly to our understanding of phenotypic aberrations and physiological modulations during disease state [74–76]. In recent studies from our group, using integrative omics and immune phenotyping comparing different HIV disease states, e.g., EC with PLWH with cART for more than a decade of treatment, we reported critical differences linked with (a) the dysregulated energy metabolism and (b) observations of unique immune-metabolic properties of myeloid lineages related with the HIV-persistence [32, 35••, 74, 77••, 78]. We also performed extensive studies on three different cohorts from India [79], Cameroon [35••], and Sweden, with more than 500 PLWH, indicating that disrupted glutaminolysis is prevalent in PLWH_ART_ in all three cohorts (Fig. 2) that play the central role in the comorbidities such as metabolic syndrome (MetS) observed in another Danish cohort [35••, 77••]. Additionally, a seminal multi-omics analysis study contributed to our understanding of immunometabolic regulation during HIV infection in CD4^+^ T cells [80].

We also developed a context-specific system-level Genome-Scale Metabolic Model (GSMM) using global RNA sequencing data on blood cells from PLWH that had suppressive viremia by natural mechanisms (EC) or drug-induced suppression (PLWH on cART) [81]. To provide a comprehensive system-level characterization, this GSMM was also compared to HIV-negative controls. The transcriptomic analysis identified upregulation of OXPHOS as a characteristic of PLWH on cART, differentiating them from the EC group. To see the flow of metabolites through the metabolic pathways, Flux balance analysis identified alterations in the flux of several intermediates in glycolysis, including pyruvate, ɑKG, and glutamate, in PLWH on cART. The topological analysis to understand the metabolic reprogramming metabolic networks were generated for PLWH on cART, EC, and HC cohorts. The reactions’ metabolic networks and associated genes exhibited significant diverging flux among the three cohorts. Among the metabolites, ɑKG uniquely plays a central role in PLWH on cART and EC compared to HC, indicative of a role of ɑKG in PLWH. ɑKG plays an essential regulatory role in modulating the metabolism in macrophages [82] by altering immune-cell function affecting human health that can be restored through metabolic intervention [83]. Despite the recent advancement of immune aging in PLWH, we do not fully understand how HIV affects the immune system and other organs during aging. Further knowledge could help to identify persons at risk for non-infectious severe complications. Application of the system biology studies by combining several layers of omic data, a comprehensive understanding of the aging phenotype and mechanism of immune aging in the PLWH can be possible.

## Conclusion

Does PLWH on successful therapy aging faster? Several terms, like accelerated, premature, or accentuated aging, were used in defining aging in PLWH. The terms accelerated and premature aging crudely describe increased changes over time that would arise earlier and increase progressively. In contrast, accentuated aging describes an increased burden of age-related damage that occurs at the same age as the general population and is static over time [84]. HIV-associated non-AIDS conditions like cardiovascular diseases, metabolic syndrome (MetS), cancers, liver diseases, and neurocognitive diseases can be associated with advancing age, where HIV or the cART may be an additional risk factor for accentuated aging in PLWH. Low-grade persistence inflammation and macrophage activation despite successful cART may play a crucial role in premature age-associated diseases and immunosenescence in PLWH. A shift towards the systemic glutamate metabolism during prolonged cART [35••, 79, 85] may potentiate the metabolic profile at-risk for accelerated aging. The epigenetic age acceleration (EAA) was reported in PLWH without ART in all tissue [12]. However, the initiation of the treatment may slow the EAA [14••]. There is no doubt that the development of specific clinical geriatric syndromes is hastened in PLWH [84].

Given the multi-faced nature of biological aging in PLWH without any biological aging biomarkers, it is essential to design an appropriate study with an adequately matched control group that most current studies failed to address. The confounding factors like lifestyle, diet, environmental pre-disposition, pre-ART immunodeficiency, and cART itself should be considered in the study design to define the biological aging in PLWH. The development and application of systems biology and machine learning methodologies can provide a better understanding of the mechanisms and temporal dynamics of biological aging in PLWH. Applying multi-omics systems biology to define the immunometabolism and biological aging in PLWH could be beneficial to identify the mechanisms of accelerated, premature, or accentuated biological aging in PLWH if it persists.
